# Risk Transmission and Resilience of China’s Corn Import Trade Network

**DOI:** 10.3390/foods14081401

**Published:** 2025-04-18

**Authors:** Jun Wu, Jing Zhu

**Affiliations:** 1 College of Economics and Management, Nanjing Agricultural University, Nanjing 210095, China; 2 Guangzhou Institute of Science and Technology, Guangzhou 510540, China

**Keywords:** corn trade, network resilience, resistance capacity, recovery capacity, risk propagation, food security

## Abstract

The global corn trade is an important pillar of the agricultural economy, but its supply chain is vulnerable to geopolitical conflicts, climate change, and market volatility. As one of the major importers of corn in the world, China has long relied on the United States and Ukraine, and the risk of import concentration is prominent. The complicated international situation intensifies the external uncertainty of China’s import supply chain. This study utilized bilateral trade data from 2010 to 2023 and employed advanced methodologies including complex network modeling, network index quantification, and simulation analysis to assess the impacts of external risks from major trading partners on China’s corn import system and trace risk transmission pathways. The research objectives focused on examining the structural evolution of China’s corn import trade network over the past decade, evaluating its resilience against external shocks, and identifying the critical roles played by key node countries in risk propagation mechanisms. The results showed that the resilience of China’s corn import trade network had been enhanced in recent years and that the complementarity of planting cycles in the Northern and Southern Hemispheres and the adjustment of trade structure caused by the Russia–Ukraine conflict had improved its risk resistance. The United States, France, Romania, and Turkey were key intermediate nodes in risk transmission due to their geographical advantages and trade hub statuses. The risk transmission path presented regional heterogeneity. China should strengthen trade with countries in the Southern Hemisphere and built a more stable import system by taking advantage of complementary resource endowments and growth periods. Bilateral agreements with transit countries could ensure security of supply. Reserve centers and modern logistics infrastructure should be built in key areas. In addition, platforms such as the Regional Comprehensive Economic Partnership could promote harmonized standards and digital support for corn trade, and regional financial instruments and supply chain optimization could have balanced risks.

## 1. Introduction

### 1.1. Research Background

Corn, as one of the world’s three main grains, plays a critical role in global agricultural trade networks. It serves as a core source of human rations, animal husbandry feed, and a cornerstone of industrial chains such as in biofuels and starch processing. However, the global trade of corn is susceptible to various risk factors, such as geopolitical tensions, climate change, and market volatility, which can disrupt supply chains and affect price stability [1,2]. To mitigate these risks, resilience mechanisms such as trade diversification, strategic reserves, and international cooperation are essential to maintaining the stability of the global corn trade.

Within this broader framework, the role of individual regions and countries in the global corn trade becomes particularly significant. China, for instance, plays a pivotal role in shaping global corn trade dynamics [3]. With its large population base, China’s demand for corn spans across food supply, the feed industry, and industrial processing. Since 2020, China’s corn imports have consistently exceeded quota limits, this trend underscores China’s growing reliance on the global corn market [4]. In addition, China’s corn imports are highly concentrated, with the United States and Ukraine accounting for more than 90 percent of its total imports in most years. In 2023, Brazil replaced the United States and Ukraine to become China’s largest source of imports [5]. This high level of import concentration limits China’s flexibility in adjusting its trade policies and highlights the need for diversification to enhance resilience.

At present, the global economic and political situation is increasingly complex and volatile, and the China–US trade tensions, the COVID-19 epidemic, the Russia–Ukraine conflict, and other incidents have increased the uncertainty of trade relations. These fluctuations in the international market may not only bring major risks to China’s corn import network and threaten the security of China’s corn supply, but also force China to adjust its trade pattern and enhance its ability to cope with external risks. So, how has the structure of China’s corn import trade network changed in the past decade? Is the network more resilient in the face of external risks? Which countries are key nodes in the risk transmission process? Understanding these issues is crucial for China to effectively respond to changes in international corn trade and ensure a stable corn supply.

### 1.2. Literature Review

#### 1.2.1. External Risks and Corn Trade

Research specifically focused on China’s corn imports is limited, mainly because the main source of China’s corn supply is domestic production, which reduces the focus on import trade. However, the emergence of unusual phenomena such as “price inversion” and “foreign corn entering the market and domestic corn entering storage” has attracted great attention from scholars [6]. The subsequent reform of China’s corn reserve policy also prompted the study of the influence of this policy on corn imports. China’s corn imports have continued to grow in recent years, especially after the signing of the US–China Free Trade Agreement. The Phase I trade agreement and the outbreak of the conflict between Russia and Ukraine have highlighted the challenges facing China’s corn imports [7,8].

From a global perspective, there are many people concerned about the corn trade [9,10]. In particular, with the COVID-19 epidemic and the Russia–Ukraine conflict as their starting points, researchers have analyzed price increases [11], network resilience [12,13], supply chain vulnerability [14], etc. These studies generally agree that these external risks raise market prices for agricultural products, reduce cyber resilience, and increase supply chain vulnerability. As an important link in the global corn trade network, China’s resilience is very important to maintaining the stability of the global trade network. However, existing studies lack a quantification of the resilience of China’s corn import trade network, which is insufficient to understand China’s ability to cope with risks. The conclusions of this paper can fill this gap.

#### 1.2.2. Cascade Effect and Resilience of Network

As a system, trade networks usually go through four typical stages when facing risks or crises: the preparation stage, degradation stage, recovery stage, and post-adaptation stage [15,16]. The degradation stage represents the impact of external risks on system performance and reflects the ability of the system to resist risks [17], while the recovery stage measures the ability of the system to recover to its initial state [18]. These two stages together constitute the main reaction process to external shocks. For the degradation stage, existing studies usually simulate external risks by randomly or selectively removing nodes or reducing their performance [19,20,21,22]. The resistance of the network to different risk sources is assessed by the change (or degradation) in network performance before and after node removal. When nodes are selectively removed, studies often use the weighting of nodes (i.e., the volume of trade) to determine which countries to remove. The basic assumption is that countries with greater trade volumes (and therefore greater weight) have greater impacts on the network, are more suitable as source countries of risk, and are preferentially removed from the network. While this approach is effective, it is not comprehensive. For example, a country at the center of a trade network, acting as an intermediary or bridge between multiple countries, may have a greater impact on the network even if its trade volume is not the largest.

When risk spreads in a trade network, there will be a cascade effect [23,24,25]. A cascade effect means that the failure of a node or connection in the system spreads through dependency relationships, triggering a chain reaction and leading to a wider range of systemic collapse [26], which is a natural threat to network resilience. This cascading phenomenon is mainly due to the presence of intermediate countries in trade networks. When intermediate countries experience import shortages, they may choose to restrict exports, thereby transferring the risk to other countries. Studies of cascading effects have covered various fields, including power grids [27], urban transportation networks [28], agricultural trade networks [29], and financial networks [30]. Some of these studies have used suspicious-infection-recovery (SIR) models and bootup penetration (BP) models to describe the risk transmission process, which assume that changes in node state depend on a certain probability [31] or a given threshold. However, An et al. (2021) believed that there would be a certain proportion of risk propagation regardless of node state, so they built a non-failure cascade model applied to the oil trade network [32]. This paper believes that food security is closely related to social stability. When external risks occur, even if a country is in a state of food security, it will increase domestic reserves and reduce exports to pass on the risks. Therefore, this paper draws on the research of An et al. (2021) and uses the core idea of the non-failure cascade model [32].

Most studies on the recovery phase of trade networks rely on theoretical frameworks and qualitative analysis, with limited quantitative evaluation. A few scholars incorporate the recovery phase into resilience assessments, but their recovery rules are often oversimplified. Yu Y (2023) assumed that the faulty node recovers randomly or according to the degree to which it was affected, which cannot accurately reflect the recovery process in real scenarios [33]. Yi L. and J. L. Wang (2024) believe that risk sources will take corrective measures to restore exports in that year [34]. This assumption is reasonable for resource-based products such as oil, but does not apply to agricultural products subject to seasonal production cycles. Countries in tropical and subtropical regions can usually grow corn in more than one season, while countries in temperate regions usually only grow corn in one season. Therefore, if temperate countries reduce production or exports due to external risks, they are unlikely to return to pre-risk export levels in the same year, and the global maize trade gap must be filled by other countries.

To sum up, there are the following gaps in the existing studies on the resilience of trade networks: First, the existing studies on resistance capacity have relatively simple selection criteria for risk source countries. Second, existing studies on recovery capacity do not set recovery rules appropriate for agricultural trade. Therefore, this study has made two improvements: One is to identify key countries from two dimensions. Countries with a large trade scale and countries in a key position in the trade network are selected as risk source countries by using their weighted out-degrees and closeness centralities. The second is to set up recovery rules suitable for agricultural trade. That is, first of all, we determine the maize planting characteristics according to the geographical location of the risk source country. If the country is in a single-season planting climate, then we set the situation so that other countries’ exports will increase; if the country is multi-cropping, then we set the situation so that the country’s exports will increase.

When the probability of external risks increases significantly, it is of great significance to understand the elasticity of China’s corn import trade network and identify the weak links and key countries in the risk transmission process. Therefore, this paper collected bilateral trade data from 2010 to 2023; built a global corn trade network; simulated the resistance and recovery capacity of China’s corn import trade network under two scenarios of export volume reduction and export volume increase; comprehensively evaluated the resilience of the trade network, especially the changes before and after the Russia–Ukraine conflict; and analyzed the risk transmission path. The rest of the paper is arranged as follows: the second part introduces the method and data, the third part contains the simulation results and analysis, the fourth part is the discussion of the results, and the fifth part is our conclusions and suggestions.

## 2. Data and Methods

### 2.1. Data Sources and Data Processing

Constructing the corn trade flow matrix was critical for assessing the resilience of the global corn trade network. In this study, bilateral trade data from 2010 to 2023, sourced from the FAOSTAT database of the United Nations Food and Agriculture Organization (FAO), were used. The countries involved in the corn trade are represented as nodes in the network, and their trade relations form the edges. The weight of each edge was determined by the trade volume between the countries, resulting in a directed and weighted corn trade network with a long time series. This study’s data begin in 2010, as China became a net importer of corn that year, marking a shift in the supply–demand pattern. A continuous and comparable time range is essential for understanding the resilience of China’s corn import trade network, allowing a deeper insight into potential challenges and enabling China to better respond to future issues.

In the processing of national nodes, the corn trade flows from Hong Kong, Macau, and Taiwan were consolidated into China, overseas territories without independent sovereign governments were incorporated into their parent country, and economic unions were deleted, retaining only independent countries. Furthermore, if the trade volume between any two countries was too small, their potential impact would have been very limited; thus, country pairs with bilateral trade volumes below 500 tons were excluded. Simultaneously, considering the usual discrepancies in import and export data, this paper was based on the following assumptions and existing research methods [35,36]: (1) if the bilateral trade volume between the same country pair was inconsistent, then the average of the two was taken as the bilateral trade volume in this paper; (2) if the export data of one country to another country was missing, then this paper replaced the import data of the latter with the former.

In addition, to address possible biases from data sources, this study combined data from multiple sources. Specifically, in addition to the FAOSTAT database, this paper also collected detailed trade statistics from the national customs and trade authorities of major trading nations to ensure granularity and accuracy. In this paper, these data were thoroughly cleaned and preprocessed to a uniform format, missing values were processed by interpolation, and statistical methods such as Z-scores were used to detect outliers. We then cross-verified the data across sources to ensure consistency and reliability and reconciled the differences by averaging or weighted averaging based on data confidence. This paper considered that the above steps can reduce the possibility of bias due to data limitations.

### 2.2. Method

#### 2.2.1. Methods of Complex Network Analysis

Complex networks describe a wide range of systems in nature and society, and like a map or diagram, networks capture the fundamental features of the system [37]. This approach provides a natural framework for the study of complex systems, allowing us to see how individual components are connected and how these connections influence the overall behavior of the system [38]. According to our method, the global corn trade network was represented as G = ($V_{i}$, $V_{j}$, A, W), where $V_{i}$ = [$v_{i}$] (i = 1, 2, …, n) represents the exporting countries and $V_{j}$ = [$v_{j}$] (j = 1, 2, …, n) represents the importing countries. The union of exporting and importing countries formed the set of nodes in the global corn trade network. A is the directed adjacency matrix of the global corn trade network, where $a_{ij}$(t) represents whether country $V_{i}$ exports corn to country $V_{j}$ in year t. If this trade exists, $a_{ij}$(t) = 1; otherwise, $a_{ij}$(t) = 0. W is the weight matrix for the global corn trade network, where $w_{ij}$(t) represents the trade volume of corn exported from country $V_{i}$ to country $V_{j}$ in year t.

To clearly illustrate the historical evolution of the global corn trade network, this study visualized the global corn trade networks for the years 2010, 2021, 2022, and 2023 using Gephi 0.10.1 software. The visualizations for 2021–2023 were designed to observe the impact of the Russia–Ukraine conflict on the global corn trade pattern and China’s corn import pattern. In these visualizations, nodes represent countries, edges represent trade links between countries, and the thickness of the edges reflects the trade volume, with thick lines indicating larger trade volumes. Arrows indicate the direction of trade relationships.

#### 2.2.2. Method of Cascade Effect Simulation Analysis

In this study, the resilience of China’s corn import trade network was quantified by a simulation method. The basic logic is this: If reducing corn exports from risk source countries (or increasing corn exports from other countries) does not significantly reduce (or increase) China’s corn imports, this indicates that China’s corn import network has a higher resistance (or reduced resilience) to risks from that country. Conversely, if the impact is large, it is considered to be less resistant to impact (or more resilient). To measure the size of the impact, we used the total flow traffic of the network, which can be calculated by Formula (1) [39]:

(1) $$W=\sum_{E}w(v,u)$$

Here, W represents the total flow of China’s corn import trade network, w(v,u) represents the bilateral trade volume between importing country u and exporting country v, and E represents the set of all edges in China’s corn trade network.

**Figure 1:**
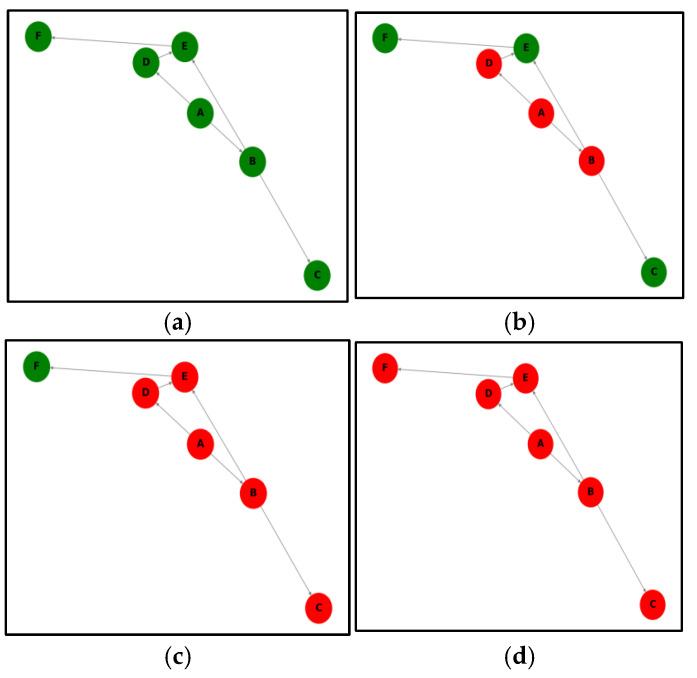
An illustration of the cascading effect in a trade network. (**a**) The initial state when no external risks have occurred, where A to F represent six countries and the arrows indicate the direction of exports. (**b**–**d**) A situation in which countries are affected successively after the occurrence of external risks. Data source: Compiled by the author based on available information.

Whether exports increase or decrease, many countries will be affected by the cascading effect. The cascade effect refers to an initial event that sets off a series of chain reactions that lead to other events in the system. For instance, if country A experiences a 10% reduction in corn exports due to external risks, this will directly affect countries B and D, which import corn from A (Figure 1b). As the imports of B and D decrease, their exports will also decline, further affecting countries C and E (Figure 1c). This cascading process continues until the effect dissipates or no further countries are impacted. The cascade effect thus provides a measure of the network’s resistance capacity. Additionally, the impact on countries B and D differs from that on countries C, E, and F. The former represents direct effects (i.e., countries with direct trade relations with the affected country), while the latter represents indirect effects (i.e., risk is transmitted via intermediary countries).

It is important to note that the reduction in imports by downstream countries is influenced by their dependence on upstream countries. The greater the dependence, the more significant the reduction in imports. The formula for calculating dependence is presented in Formula (2) [40]:

(2) $${dependence}_{v,u}=\frac{{import}_{u,v}}{{export}_{v}}$$

Here, ${dependence}_{v,u}$ indicates the degree of dependency of importing country u on exporting country v, ${import}_{u,v}$ indicates the quantity of corn imported by u from country v, and ${export}_{v}$ indicates the total amount of corn exported by country v. Given the common presence of self-loops in trade networks, this paper assumed that each country’s import loss would only be transmitted once.

#### 2.2.3. Method of Indicator Quantification

In this paper, the weighted out-degrees and closeness centrality of countries were calculated in order to screen out important countries as risk sources. Table 1 presents the calculation methods and explanations for weighted out-degree and closeness centrality values.

The existing external risk impact research uses a variety of methods, including econometric models [41,42] and equilibrium analysis [43,44]. In this paper, the dynamic analysis of China’s corn import elasticity was carried out by using complex network modeling and simulation, and the network structure was visualized, key nodes were identified, and the elasticity was quantified by index and cascade effect simulation. At the same time, we also incorporated sector-specific recovery rules, which were essential in order to capture real-world agricultural trade dynamics. While other approaches have their merits, they may not fully address complex network interdependence and adaptive behavior.

### 2.3. Evaluation Framework for Trade Network Resilience

#### 2.3.1. Complex Network Modeling of China’s Corn Import Trade System

A complex network model was used to construct the global corn trade network, and China’s corn import trade network was extracted from it.

#### 2.3.2. Identification of Risk Sources in Global Corn Trade System

Existing research suggests that agricultural trade networks typically exhibit structural features such as small-world characteristics, power-law distributions, export concentration, and dispersed imports [45,46,47,48]. These network structures tend to show strong resistance to random external risks (e.g., natural disasters) but exhibit weaker recovery capabilities. In contrast, these networks may have lower resistances to targeted external risks (e.g., wars), although their recovery capabilities may be typically stronger. Given that random risks are usually unpredictable and targeted risks often have a more significant impact, this study focuses primarily on analyzing the effects of targeted external risks.

To assess the impact of targeted external risks, it is crucial to identify risk source countries. Unlike previous studies, this research evaluated importance from two dimensions: trade volume importance and network position importance. Generally, countries with higher weighted out-degree values tend to have larger export volumes, meaning a reduction in their exports is likely to have a larger impact on the network. Countries with higher closeness centrality are typically more centrally located within the trade network, and a reduction in their exports will have a more widespread effect. The calculation methods are shown in Table 1.

#### 2.3.3. Resilience Capacity Assessment of China’s Corn Import System

This paper quantifies the resilience of China’s maize import trade network in two aspects: resistance capacity and recovery capacity. For the resistance capacity, we first determined the risk source countries by weighted out-degree and closeness centrality, and then the total flow of China’s corn import trade network under the cascade effect was simulated and calculated, assuming a 10% reduction in exports from these countries. Finally, the total flow was compared with that without external risks to reflect the strength of its resistance capacity.

To quantify recovery capacity, this study also used a simulation approach: First, maize planting characteristics were determined based on the geographic location of the risk source country. If the country implemented single-season planting, the increase in exports of other countries was simulated; if the country had multiple crops, an increase in the country’s exports was simulated. Similarly to export reductions, export increases also exhibit cascade effects. Not only will they increase the imports of directly affected countries, but they may also raise imports for countries with indirect trade relationships.

It should be noted that export increases and decreases are not independent processes. The effect of export increases is based on the cumulative effects after export reductions. Specifically, a reduction in exports alters edge weights and modifies dependencies, meaning that the simulation of export increases was based on these updated dependencies.

#### 2.3.4. Risk Propagation Pathways in China’s Corn Import Network

We used graph theory path search technology to deconstruct transnational risk transmission paths and identify key hub nodes and potential vulnerable links in China’s import trade.

## 3. Results

In this section, the paper initially presents an analysis of the evolution of the corn trade network structure commencing from 2010. Subsequently, it computes the weighted out-degree and closeness centrality of countries to pinpoint the pivotal nations within the trade network. Following that, the paper presents a simulation to assess the resistance capacity and recovery capacity of China’s corn import trade network under the scenario of reduced exports from these key countries, with a particular emphasis on the backdrop of the Russia–Ukraine conflict. Ultimately, the paper carries out a more in-depth analysis of the risk transmission pathways and identifies the intermediate countries participating in the risk propagation process.

### 3.1. The Evolution of the Structure of the Global Corn Trade Network

**Figure 2:**
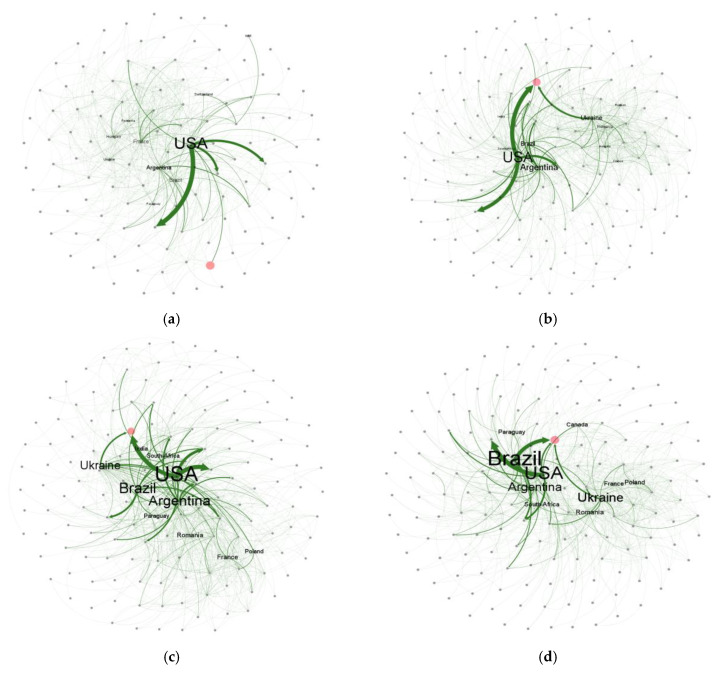
The evolution of the global corn trade network. (**a**) The global corn trade network for 2010, (**b**) the global corn trade network for 2021, (**c**) the global corn trade network for 2022, and (**d**) the global corn trade networks for 2023. Notes: (1) The red node represents China. (2) The top 10 countries in the global maize trade network with the highest weighted out-degree are labeled to identify the major exporters. Data source: Obtained from graphs created using Gephi 0.10.1 software.

Overall, the structure of the global maize trade network was characterized by concentrated exports and dispersed imports, and its distribution conformed to the power law (Figure 2). The structural characteristics of this trade network also supported the rationality of the research direction of this paper, that is, focusing on specific and targeted external risks and ignoring random external risks, which is more conducive to developing more efficient risk management and response strategies.

Specifically, in 2010, the United States was the absolute core of the global corn trade network, and its trade flows showed significant “unipolar radiation” characteristics, mainly from the United States to the rest of the world. The United States controlled almost the main global flow of corn, and its exports were far larger than those of other countries. By 2023, the density of the trade network had continued to increase but the “unipolar radiation” pattern led by the United States had changed. Ukraine, Brazil, and other countries had gradually risen and the global corn trade network was gradually developing from its traditional unipolar pattern to a multipolar direction. Correspondingly, the source countries of China’s corn imports increased with the expansion of the import scale, which confirmed the change in the global corn trade pattern.

The pattern of China’s corn import showed remarkable characteristics of phased evolution. A dual-nucleus supply system gradually formed between 2010 and 2021 (led by the United States and supplemented by Ukraine); this dependence path continued in the early stages of the Russia–Ukraine conflict in 2022, as shown by the lack of immediate adjustments in the market shares of the main suppliers. But, in 2023, China’s corn import trade underwent structural adjustment: the main source countries of its imports became Brazil, the United States, and Ukraine, and Brazil surpassed the United States and Ukraine to become the largest source country of imports.

### 3.2. The Analysis of the Importance of Countries in the Corn Trade Network

**Figure 3:**
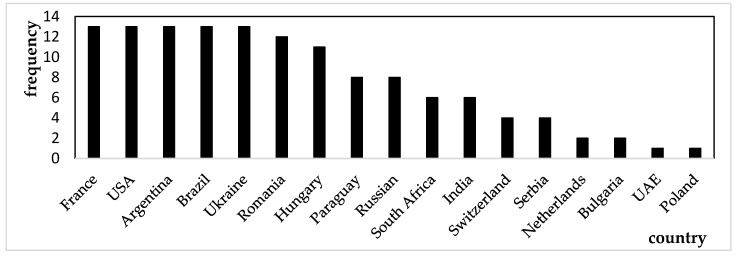
The frequency distribution of the top ten countries with the highest out-degree from 2010 to 2023. Data source: Compiled by the author.

**Figure 4:**
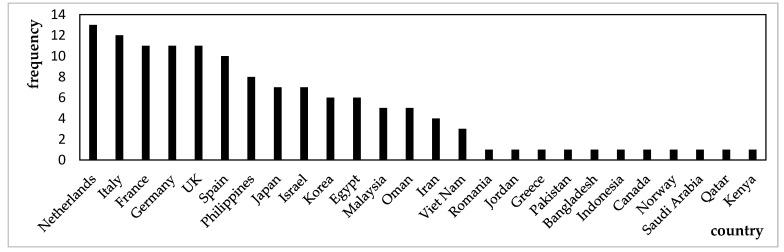
The frequency distribution of the top ten countries with the highest closeness centrality from 2010 to 2023. Data source: Compiled by the author.

This study calculated the weighted out-degree and closeness centrality of each country in the global corn trade network from 2010 to 2023, performing an annual analysis. The top ten countries with the highest weighted out-degree and closeness centrality values each year were recorded. Additionally, frequency distribution charts were created based on the frequency of each country’s appearance, as shown in Figure 3 and Figure 4.

Figure 3 and Figure 4 illustrate the countries with the largest export volumes and those occupying central positions in the global corn trade network from 2010 to 2023. Figure 3 presents a smaller group of countries, suggesting that major corn exporters, such as France, the United States, Brazil, and Argentina, had long dominated global corn exports, establishing a stable supply network. In contrast, Figure 4 shows a larger number of countries with a more dispersed distribution, reflecting shifting central positions within the global trade network. This change might be attributed to shifts in the global economic landscape, adjustments in trade relations, and fluctuations in market demand.

Although Figure 4 shows a broader distribution of countries, European nations like France, the Netherlands, and Italy consistently maintained central roles in the global corn trade network; this was likely due to their critical position in the global supply chain. Additionally, the differences between Figure 3 and Figure 4 indicate that countries with higher export volumes do not necessarily occupy central positions within the global corn trade network. Therefore, when analyzing the network, it was crucial to consider both the export volumes and the geographical locations of countries. This comprehensive approach aided in identifying which countries’ export reductions would have a more significant impact on both the global corn trade network and China’s corn import supply.

### 3.3. Resistance Capacity of China’s Corn Import Trade Network

**Figure 5:**
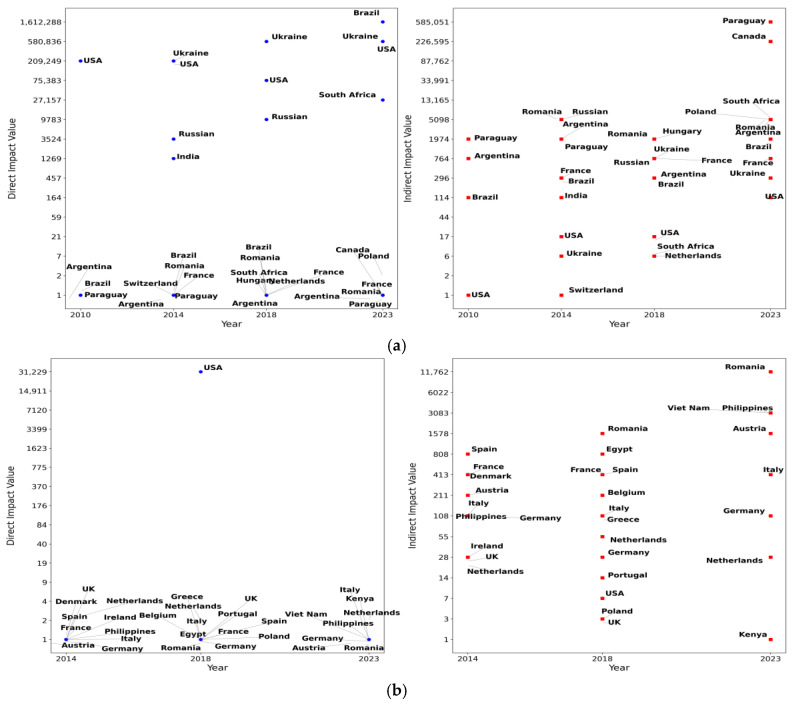
The impact of reduced exports from the top ten countries by closeness centrality and weighted out-degree in certain years on China (tons). (**a**) The direct impact and indirect impact of export reductions in risk source countries selected based on weighted out-degree; (**b**) the direct impact and indirect impact of export reductions in risk source countries selected based on closeness centrality. Notes: (1) Because the effects vary widely from country to country, these values are logarithmic in order to spread the data more evenly across the chart. The value of the ordinate is the value after taking the logarithm. (2) In 2010, the risk source countries selected according to closeness centrality had neither direct nor indirect trade relations with China, so (**b**) only shows the simulation results for 2014, 2018, and 2023. Data source: Obtained by the author.

This study simulated the direct and indirect impacts on China’s corn imports when the export volume of the top ten countries with the highest closeness centrality and weighted out-degree values decreased by 10%. The results are presented in Figure 5.

The results indicate that China’s corn import trade network demonstrates robust resilience when faced with reduced corn exports from countries with high closeness centrality. This resilience is reflected in two main aspects. First, the impact is delayed. The reduction in corn exports from countries with high closeness centrality does not immediately affect China’s corn imports. Specifically, in 2010, the reduction in exports from these countries did not result in any direct or indirect impact on China’s corn imports. It was only after 2014, as global trade network density increased and trade links expanded, that indirect impacts began to manifest. However, the lack of direct trade relationships allowed this indirect impact to provide a buffering effect for China’s corn imports. Second, the magnitude of the impact is relatively limited. Specifically, when countries with high closeness centrality reduced their corn exports, the reduction in China’s corn imports was primarily in the range of 100 to 1000 tons. Compared to the overall scale of China’s corn imports, this reduction is negligible and unlikely to cause significant disruption to the import structure or volume.

Similarly, the reduction in corn exports from larger exporting countries did not significantly affect China’s corn imports, indicating that China’s corn import trade network had strong resilience in dealing with external risks from these countries. However, two noteworthy trends gradually emerged: First, the direct influence of the United States, Ukraine, and Brazil on China’s corn imports increased significantly. Since 2014, the direct impact of reduced exports from these countries on China’s corn imports increased by 1347.29%, 445.84%, and 1,612,287%, respectively. Second, the indirect impact of declining exports from Argentina, Canada, Paraguay, and other countries on China also expanded. For example, in 2014, a 10% reduction in Argentine corn exports only resulted in a 783.11 ton reduction in Chinese imports, while, by 2023, the reduction in Argentine corn exports resulted in a 3279.04 ton reduction in Chinese imports.

In addition, the results of Figure 5 also show a certain geographical concentration: the countries that have a significant impact on China’s corn imports are mainly concentrated in the Northern Hemisphere. This phenomenon may be due to the Northern Hemisphere countries’ stronger ability to export to markets, as well as closer geographical relationships.

### 3.4. Recovery Capacity of China’s Corn Import Trade Network

The resilience analysis revealed that countries with significant impacts on China’s corn imports, such as the United States and Ukraine, were predominantly located in temperate regions. These countries typically engage in single-season corn planting. As a result, when these countries experience a reduction in exports due to external risks, they are unable to recover production to pre-risk levels within the same year, requiring other countries to fill the trade gap. To explore this dynamic further, this study simulated a scenario where the U.S. and Ukraine’s corn exports decreased by 10%, while corn exports from Brazil, Argentina, and South Africa increased by 50%. These countries were selected because they were located in tropical and subtropical regions with favorable conditions for corn growth, allowing for multi-season planting (e.g., Brazil can grow both summer and winter corn), leading to higher yields. These countries had the potential to become major global corn exporters. The simulation results are presented in Table 2.

According to these results, in the current international corn trade pattern, the resilience of China’s corn imports has continued to increase, which is shown as the total impact shows a downward trend. In particular, in 2023, the increase in Brazilian corn exports more than compensated for the negative impact of lower exports from the United States and Ukraine. But, beyond that, even a 50% increase in corn exports from other Southern Hemisphere countries would not make up for the shortfall in corn from the United States and Ukraine. For example, in 2023, the decrease in US exports led to a direct corn decrease in China of 565,888.85 tons, while the increase in corn from Argentina was only 14,797.43 tons. This showed that, in China’s corn import supply chain, the United States, Brazil and Ukraine formed a three-pillar trend; the external risk of a certain country could be made up by the other two countries, and the recovery ability of China’s imports was strong, but other countries except these three countries could not make China’s corn imports recover greatly.

### 3.5. The Analysis of the Resilience of China’s Corn Imports Under the Background of the Russia–Ukraine Conflict

Judging from bilateral trade data, Ukraine was once an important pillar of China’s “dual-core” supply system for corn imports. From 2019 to 2021, Ukraine exported 6.29 million tons of corn to China on average annually, accounting for 37.2% of China’s total imports. After the outbreak of the conflict between Russia and Ukraine, logistics transportation channels were blocked. On the one hand, this was reflected in exports: the military blockade of major ports such as Odessa directly led to the basic stagnation of Ukrainian grain exports. On the other hand, this was reflected in imports: the supply chain of Ukraine’s agricultural means of production had been disrupted, resulting in a decrease of about 11% in the maize sown area in 2023 compared with the same period in the previous year. In view of the close relationship between the Russia–Ukraine conflict and China’s corn imports, this paper further calculated the resilience of China’s corn import trade network in 2021–2023 in this context, in order to analyze the impact of the Russia–Ukraine conflict. The results are shown in Table 3.

Since the outbreak of the conflict between Russia and Ukraine, the resilience of China’s corn import trade network has increased: the overall impact of reduced exports on China’s corn imports has continued to decrease. When the United States and Ukraine, two traditional corn exporting countries, reduced their exports, the reduction in China’s corn import trade flow was significantly smaller than in the past, indicating that the trade network’s resistance ability was substantially enhanced when dealing with direct trade disruptions or indirect impacts caused by trade transmission. It is particularly noteworthy that, after the conflict between Russia and Ukraine, the positive performance of Brazil and South Africa in corn exports effectively compensated for the adverse impact of the reduction in exports from the United States and Ukraine; especially in 2023, this compensating role was more prominent, and, thus, the resilience of China’s corn import trade network was greatly improved.

### 3.6. Risk Transmission Pathways in China’s Corn Imports

#### 3.6.1. General Analysis of Risk Transmission Paths

Table 4 presents the average length of risk transmission paths. The change in this indicator is small, consistent with the expectations of the “small world” theory. According to the “small world” theory, in complex network structures, most nodes are connected through short paths. Between 2010 and 2023, although the connectivity within the global corn trade network changed, the average path length remained low, demonstrating typical “small world” characteristics.

**Figure 6:**
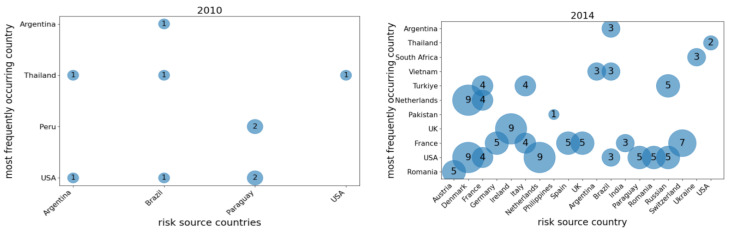

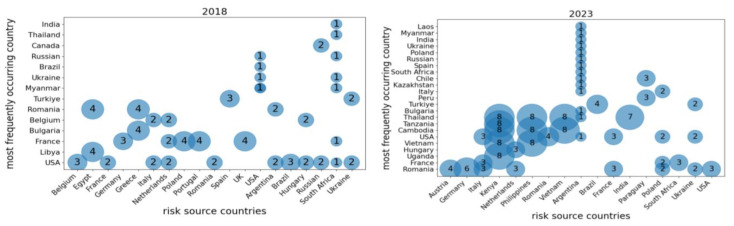
In the process of risk transmission, these are the most frequent countries and their frequencies of occurrence. Notes: (1) Risk source countries selected according to weighted out-degree in 2010: Argentina, Brazil, Paraguay, and the USA; risk source countries selected according to weighted out-degree in 2014: Argentina, Brazil, France, India, Paraguay, Romania, Russia, Switzerland, Ukraine, and the USA; risk source countries selected according to weighted out-degree in 2018: Argentina, Brazil, France, Hungary, Netherlands, Romania, Russia, South Africa, Ukraine, and the USA; risk source countries selected according to weighted out-degree in 2023: Argentina, Brazil, France, India, Paraguay, Poland, Romania, South Africa, Ukraine, and the USA. (2) Risk source countries selected according to closeness centrality in 2014: Austria, Denmark, France, Germany, Ireland, Italy, Netherlands, Philippines, Spain, and the UK; risk source countries selected according to closeness centrality in 2018: Belgium, Egypt, France, Germany, Greece, Italy, Netherlands, Poland, Portugal, Romania, Spain, and the UK; risk source countries selected according to closeness centrality in 2023: Austria, Germany, Italy, Kenya, Netherlands, Philippines, Romania, and Vietnam. (3) The numbers in the bubbles represent the number of times a country was present during the risk transmission process. Data source: Obtained by the author.

In addition, the paper also documented the countries where risk transmission paths occur frequently (Figure 6); when countries with high closeness centrality became risk sources, the most frequent countries appearing in the risk transmission paths included the United States, France, and Romania. On the other hand, when countries with high weighted out-degrees acted as risk sources, the most frequent countries in the risk transmission paths included the United States, France, Thailand, Turkey, and Romania. This showed that these countries were not only major countries in global corn production and exports, but also “key nodes” in the global corn supply chain. Corn production and trade policy changes in these countries directly affected the global corn supply chain, especially when their exports were affected by risks (such as natural disasters, economic sanctions, etc.), which may pass risks through other countries and have a ripple effect on the Chinese market.

#### 3.6.2. Detailed Analysis of Risk Transmission Paths: A Case Study of 2023

To further analyze the risk transmission paths, this study used 2023 as an example and presented the ten most significant risk transmission paths affecting China’s corn imports, as shown in Table 5. The numbers in the table represent the reduction in China’s corn imports.

The external risks directly transmitted from the United States and Ukraine were the greatest uncertainties impacting China’s corn import security. In contrast, the risk transmission paths from Argentina, Paraguay, and other South American countries needed to be transmitted to China through the United States, which weakened their impact on the Chinese market to a certain extent. In contrast, the risk transmission ability of Asian countries to China was lower, and their final impact values were significantly smaller than those of South America, Europe, and the United States, which might have been caused by their small economic size and risk diversification effect. The economies of Asian countries such as the Philippines and Vietnam were small, their risk transmission paths involved more transit nodes, and risks were gradually dispersed and weakened in the transmission process. In addition, the risk transmission path of Asian countries was longer and was concentrated in Southeast Asia, showing a strong regional economic network characteristics. For example, the Philippines and Vietnam both passed through Cambodia and Thailand.

In summary, the risk transmission paths and the intensity of their impact on China from South American and Asian countries showed significant differences. South American countries relied on the U.S. as a trans-shipment hub for risk transmission, while Asian countries primarily exhibited regionalized supply chain structures in their risk transmission paths.

## 4. Discussion

The density of the global maize trade network is increasing, and the trade pattern is shifting from single-pole radiation to multi-pole, which reflects the gradual diversification of the global supply chain. In the past, the global corn trade was almost entirely dominated by the United States, forming a “unipolar radiation” pattern centered on the United States. However, with the rise of emerging corn producers such as Ukraine and Brazil, the global corn market has gradually transformed from a single US-dominated structure to a more diversified multipolar pattern. This trend is not only reflected in the corn trade, but also in other agricultural products [6,7,49]. This change is likely to be closely related to the diversification of global agricultural markets, the diversification of exports driven by geopolitical factors, and the rapid development of emerging market countries in agricultural production, technological innovation, and market expansion [50,51].

Diversification is certainly conducive to enhancing the resilience of corn trade networks, especially in the face of global market uncertainties, and can effectively reduce dependence on a single source of supply and enhance the resilience of the overall supply chain. However, the role of certain core countries cannot be ignored. When crises occur in these key countries, global trade may be seriously affected [52,53]. This is similar to what has been found in studies on the resilience of trade networks for other agricultural products [52]. This paper screens the key countries in the global corn trade from the perspectives of export scale and network status and points out that, when countries with large export scales encounter external risks, the direct impact on China’s corn imports is more significant, especially from the United States and Ukraine. However, this situation changed in 2023, which may be related to the outbreak of the Russia–Ukraine conflict. The Russia–Ukraine conflict has led to changes in the structure of China’s corn import trade network, forming a multi-level alternative network. This structural shift suggests that the geopolitical crisis has restructured the spatial distribution of specific commodity flows [54,55,56]. However, not all external risks can force importing countries to build multidimensional buffers; the impact of external risks is heterogeneous, political risks can have a positive impact on agricultural trade patterns, and economic instability can inhibit countries’ agricultural trade [57].

In terms of risk transmission, constraints on logistics and shipping routes, dependence on the financial system, geopolitical tensions, and other factors collectively affect the paths of risk transmission. For example, risk transmission routes from South American countries such as Argentina and Paraguay usually need to reach China through the United States. This phenomenon can be explained by two aspects: On the one hand, due to the few direct shipping lines from South American countries to China and the high transportation cost [58], many goods from South American countries need to pass through US ports before being transferred to China. On the other hand, these South American countries have long maintained close trade ties with the United States [59,60]. By investing in South American countries, American multinational companies have deeply integrated their export systems into the global economic network led by the United States [61].

The paths of risk transmission from Asian countries illustrate the complexity of the combination of geopolitical and economic dependence. Although these countries have some geopolitical tensions with China (such as the South China Sea disputes, etc.), from an economic perspective, they are highly dependent on the Chinese market, especially in terms of agricultural exports, as highlighted by Nelson Villoria (2014), who found that a 50% reduction in Chinese demand could lower food prices in developing Asian countries by 1.27% [62]. This economic dependence creates dual-channel risks: geopolitical shocks, such as military exercises in contested waters, disrupt supply chains, and reduce FDI inflows [63]. Conversely, trade volatility amplifies vulnerabilities: for instance, Vietnam’s 2016 anti-China protests triggered a USD 800 million drop in agricultural exports to China [64], underscoring how political friction directly impacts trade flows. These dynamics form a “risk feedback loop”: geopolitical instability reduces Chinese imports, causing price collapses in exporter economies, which in turn weakens their resilience to future shocks.

Although this study has obtained some meaningful conclusions, there are still some limitations. First of all, other screening criteria for the countries of origin of risk were not taken into account, for example, countries in the structural hole of the trade network, countries in the same or different communities with China [65,66], and countries with good international relations with China. The selection of countries through these criteria can further refine and improve the key countries in the global corn trade network, and more comprehensively identify the key countries in the global corn trade network and their role in risk transmission.

Secondly, although the method used in this study can effectively capture the topology of the network and the risk transmission paths, the model itself has a certain simplification. For example, when simulating risk transmission, it is assumes that risk transmission is mainly carried out through the direct and indirect links of trade relations, and the potential impact of other non-trade factors (such as policy adjustments, market expectations, etc.) on risk transmission is not fully considered [67,68,69].

Finally, the analysis of risk transmission paths in this study mainly focuses on the one-way transmission of risk from exporting countries to importing countries, but does not fully consider the interactions between importing countries and the reverse impact of importing countries on exporting countries [70,71,72]. In actual international trade, this two-way or multidirectional risk transmission can be more complex and have different effects on the resilience of the network.

## 5. Conclusions and Recommendations

### 5.1. Conclusions

In light of the increasing uncertainties in the current economic and trade environment, the risk of external threats to China’s corn import trade is rising. Based on bilateral trade data, this paper quantifies the resilience of China’s corn import trade network, analyzes the path of risk transmission, and mainly draws the following conclusions:

- China’s corn import trade network has demonstrated enhanced resilience against external risks from key exporting countries, primarily attributed to the complementary planting cycles between the Northern and Southern Hemispheres. This structural improvement was further accelerated by the Russia–Ukraine conflict, which reshaped global trade dynamics.
- Our analysis identifies the U.S., France, Romania, and Turkey as critical intermediaries in China’s corn import risk transmission network, leveraging their geographical proximity, trade network centrality, and export advantages.
- Regional risk transmission paths vary: South American risks transit through the U.S., while Asian risks propagate within regional networks with limited cross-regional impact.

### 5.2. Policy Recommendations

Combined with the research in this paper, the resilience of China’s corn import trade network can be improved in the following aspects in order to cope with possible external risks in the future:

- Strengthening trade cooperation with Southern Hemisphere countries: China should actively enhance trade cooperation with Southern Hemisphere countries such as Argentina and South Africa. These countries possess significant natural resource advantages and considerable room for expanding agricultural production. By fostering bilateral cooperation, China can encourage these countries to expand their corn cultivation, optimize their planting techniques, and boost their export capacity, thereby securing a more stable and substantial supply of corn.
- Mitigating risk transmission effects through focused engagement with intermediary countries: Intermediary countries play a pivotal role in the propagation and diffusion of risks. Therefore, focusing on intermediary countries is critical to ensuring the stability of China’s corn imports. On the one hand, China should actively negotiate bilateral agreements with intermediary countries, setting clear annual supply targets, establishing flexible adjustment mechanisms, and ensuring export volumes to China during emergencies (such as natural disasters or international conflicts). On the other hand, China should create regional supply buffer zones with intermediary countries. Strategic reserve centers should be established in key areas, such as U.S. border regions, French border zones, and in proximity to Turkey and the Middle East, specifically for the storage of corn and its processed products. Additionally, logistics parks should be set up at critical ports and railway junctions, integrating warehousing, processing, packaging, and export functions. China should also invest in modern unloading equipment and warehousing facilities at ports like the Port of Marseille in France, the Port of Le Havre, and Black Sea ports in Turkey to improve logistics efficiency.
- Optimizing the internal network structure of the Asian region: As risk transmission within Asian countries primarily occurs through regional internal networks, imbalances within these regional structures can lead to the aggregation of localized risks. To address this, China can enhance the role of existing regional trade agreements. For instance, within the RCEP framework, corn trade can be prioritized, and unified regional regulatory standards (such as for quality, transportation, processing, etc.) should be established to reduce trade friction and improve trade efficiency. Additionally, regional trade support platforms should be developed. By leveraging digital tools (e.g., electronic trading platforms), China can create a regional corn trade market that offers supply–demand data, transaction matching, price transparency, and other services to reduce transaction costs. Regional financial instruments and risk-hedging mechanisms can also be developed. For example, China could establish a corn futures exchange or trade insurance system within the region, helping regional traders and farmers manage price volatility.

### 5.3. Research Suggestions for Future Studies

At least the following three points can be further extended out from this paper:

First, this study generally describes the external risks suffered by a country without classifying them. However, the impact of different risks may be different. For example, political risks spread in the supply chain through tariffs, sanctions, export restrictions, and other means, leading to supply interruption or supplier selection changes; short-term factors such as natural disasters will affect production in some countries, leading to supply uncertainty in the short term for major importing countries. The global health crisis affects global trade by impeding the movement of people, restricting international transport, and adjusting trade policies, and countries may face supply instability, transport delays, and price increases in the short term. Different types of risks affect the corn trade network through different channels, and understanding how these risks spread through the global supply chain is critical to ensuring the stability of the corn supply chain. Future research should aim to quantify the pass-through effects of these risks and explore how resilience mechanisms can enhance the resilience of China’s maize trade networks.

Second, this study mainly uses cascade effects to characterize risk transmission, which may not be accurate enough; for example, trade substitution effects are not considered. When an importing country imports less from a source country, it must increase imports from other source countries, and the trade substitution effect occurs. However, the size of this effect is affected by many factors, such as price and level of economic development. This means that including this mechanism in models can more accurately reflect the ability of different countries to cope with risks. Therefore, future research should focus on improving the risk communication model.

Third, the study in this paper is mainly a static analysis, and future studies can analyze the structural changes and resilience characteristics of the corn trade network by building a dynamic model. The evolution laws of the network and the changes in toughness on different time scales can be studied.

## Figures and Tables

**Table 1:** Calculation methods and explanations for weighted out-degree and closeness centrality.

	Formula	Interpretation
Weighted Out-Degree	$w_{out}(v)=\sum_{(v,u)\epsilon E}w(v,u)$ $\mathrm{where}\;w_{out}(v)\;\mathrm{represents}\;\mathrm{the}\;\mathrm{weighted}\;\mathrm{out}-\mathrm{degree}\;\mathrm{of}\;\mathrm{node}\;v;\;E\;\mathrm{denotes}\;\mathrm{the}\;\mathrm{set}\;\mathrm{of}\;\mathrm{all}\;\mathrm{outgoing}\;\mathrm{edges}\;\mathrm{of}\;\mathrm{node}\;v;\;\mathrm{and}\;w(v,u)$ represents the edge weight from node v to node u.	In international trade, a country can both export and import, which can be represented using a directed graph according to graph theory. In a directed graph, edges are represented by arrows, and the number of arrows pointing away from a vertex represents its out-degree. The weighted out-degree refers to the sum of the weights of all edges originating from a certain node. It not only counts the number of outgoing edges but also considers the weight of each outgoing edge [2].
Closeness Centrality	$C(v)=\frac{1}{\sum_{(v,u)\epsilon V}d(v,u)}$ where C(v) represents the closeness centrality of node v; d(v,u) represents the shortest path distance between node v and node u; and V represents the set of all nodes in the network.	Closeness centrality reflects the reciprocal of the average distance from a node to all other nodes in the network. It is used to measure how quickly a node can reach all other nodes in the network. Nodes with a high closeness centrality are usually located at the center of the network, with shorter paths to other nodes [2].

Data source: Compiled by the author.

**Table 2:** Recovery capacity of China’s corn import trade network (tons).

Year	Reducing Countries	Increasing Countries	Direct Reduction	Direct Increase	Indirect Reduction	Indirect Increase	Total Impact
2010	USA	Argentina	150,177.90	0	0.06	1763.99	−148,413.96
	USA	Brazil	150,177.90	0	0.06	347.45	−149,830.50
	USA	South Africa	150,177.90	0	0.06	14.39	−150,163.57
	Ukraine	Argentina	0	0	0	1959.97	1959.97
	Ukraine	Brazil	0	0	0	386.05	386.05
	Ukraine	South Africa	0	0	0	14.55	14.55
2014	USA	Argentina	102,706.87	0	8.64	3309.02	−99,406.49
	USA	Brazil	102,706.87	0	8.64	670.22	−102,045.30
	USA	South Africa	102,706.87	0	8.64	497.14	−102,218.38
	Ukraine	Argentina	96,437.30	0	75.21	3674.54	−92,837.97
	Ukraine	Brazil	96,437.30	0	75.21	675.39	−95,837.12
	Ukraine	South Africa	96,437.30	0	75.21	496.29	−96,016.22
2018	USA	Argentina	31,229.81	0	6.80	836.29	−30,400.32
	USA	Brazil	31,229.81	0	6.80	870.15	−30,366.46
	USA	South Africa	31,229.81	0	6.80	18.83	−31,217.79
	Ukraine	Argentina	292,985.53	0	0.97	929.17	−292,057.32
	Ukraine	Brazil	292,985.53	0	0.97	966.71	−292,019.78
	Ukraine	South Africa	292,985.53	0	0.97	18.51	−292,967.98
2023	USA	Argentina	565,888.85	45.46	0	14,797.43	−551,136.8764
	USA	Brazil	565,888.85	45.46	8,061,423.50	6718.78	7,502,207.971
	USA	South Africa	565,888.85	45.46	83,062.94	1339.97	−481,531.4001
	Ukraine	Argentina	544,022.40	122.34	0	16,394.76	−527,749.974
	Ukraine	Brazil	544,022.40	122.34	8,061,430.28	7464.56	7,524,750.103
	Ukraine	South Africa	544,022.40	122.34	83,062.95	1339.73	−459,742.064

Data source: Obtained by the author.

**Table 3:** The resilience of China’s corn imports network in 2021–2023 (tons).

Year	Reducing Countries	Increasing Countries	Direct Reduction	Direct Increase	Indirect Reduction	Indirect Increase	Total Impact
2021	USA	Argentina	1,982,713.20	0	0	58,585.32	−1,924,127.884
	USA	Brazil	1,982,713.20	0	0	52,951.00	−1,929,762.202
	USA	South Africa	1,982,713.20	0	827.82	263.45	−1,981,621.931
	Ukraine	Argentina	823,391.70	3989.48	0	65,077.93	−762,303.2428
	Ukraine	Brazil	823,391.70	3989.48	0	58,451.55	−768,929.6251
	Ukraine	South Africa	823,391.70	3989.48	827.82	290.88	−826,262.4776
2022	USA	Argentina	1,486,467.6	0.43	0	33,816.16	−1,452,651.87
	USA	Brazil	1,486,467.6	0.43	0	23,394.64	−1,463,073.39
	USA	South Africa	1,486,467.6	0.43	310.71	19,413.68	−1,466,743.64
	Ukraine	Argentina	526,393.85	362.07	0	37,570.24	−489,185.68
	Ukraine	Brazil	526,393.85	362.07	0	25,972.51	−500,783.41
	Ukraine	South Africa	526,393.85	362.07	310.71	21,568.76	−504,876.45
2023	USA	Argentina	565,888.85	45.46	0	14,797.43	−551,136.8764
	USA	Brazil	565,888.85	45.46	8,061,423.50	6718.78	7,502,207.971
	USA	South Africa	565,888.85	45.46	83,062.94	1339.97	−481,531.4001
	Ukraine	Argentina	544,022.40	122.34	0	16,394.76	−527,749.974
	Ukraine	Brazil	544,022.40	122.34	8,061,430.28	7464.56	7,524,750.103
	Ukraine	South Africa	544,022.40	122.34	83,062.95	1339.73	−459,742.064

Data source: Obtained by the author.

**Table 4:** The average length of risk transmission paths in certain years.

Type	Year	I	II	I	II	Year	I	II	I	II
Closeness Centrality	2010	——				2014	Austria	4.56	Italy	4.89
							Denmark	5.22	Netherlands	4.22
							France	4.33	Philippines	3
							Germany	4.89	Spain	4.89
							Ireland	5.89	UK	4.89
	2018	Belgium	3.75	Netherlands	4.25	2023	Austria	4.14	Romania	3.75
		Egypt	5.25	Poland	4.25		Germany	4.5	Vietnam	6.38
		France	3.25	Portugal	4.25		Italy	4.38		
		Germany	4.25	Romania	3.25		Kenya	9.38		
		Greece	5.25	Spain	4.25		Netherlands	4.43		
		Italy	4.25	UK	4.25		Philippines	7.38		
				USA	3					
Weighted Out-Degree	2010	Argentina	3			2014	Argentina	3.67	Romania	3.89
		Brazil	3.5				Brazil	4	Russia	4.56
		Paraguay	4.5				France	4.33	Switzerland	4.86
		USA	2.5				India	3.44	Ukraine	4
							Paraguay	4.11	USA	3.22
	2018	Argentina	3.75	Romania	3.25	2023	Argentina	3.75	Poland	4.29
		Brazil	3.5	Russia	3.75		Brazil	4	Romania	3.75
		France	3.25	South Africa	3.75		France	4.13	South Africa	3.75
		Hungary	3.75	Ukraine	3.5		India	5.25	Ukraine	4.13
		Netherlands	4.25	USA	3		Paraguay	4.75	USA	3.63

Notes: Column 1 of the table shows risk source countries, which were selected according to weighted output and closeness centrality; Column II in the table represents the average length of the risk transmission path. The “--” in the table indicates that the reduction in exports from countries with high centrality would not have had an impact on China’s corn imports. Data source: Obtained by the author.

**Table 5:** Top ten risk transmission paths affecting China’s corn imports in 2023.

Risk Source Countries	Risk Transmission Path	Impact (tons)
USA	USA → China	1,486,467.60
Ukraine	Ukraine → China	526,393.85
Romania	Romania → USA → China	11,302.82
Argentina	Argentina → USA → China	7511.78
Paraguay	Paraguay → Peru → USA → China	6191.64
Brazil	Brazil → USA → China	5174.42
South Africa	South Africa → Mexico → USA → China	4312.03
Philippines	Philippines → Vietnam → Cambodia → Thailand → China	1654.80
Vietnam	Vietnam → Cambodia → Thailand → China	1654.80

Data source: Obtained by the author.

## Data Availability

The data that support the findings of this study are openly available in the database of Food and Agriculture Organization of the United Nations at https://www.fao.org/faostat/en/#home (accessed on 1 January 2025). The database is public, free, and requires no registration.
